# Supplementary material for: A new high-voltage calcium intercalation host for ultra-stable and high-power calcium rechargeable batteries
Source: Nat Commun. 2021 Jun 7;12:3369. doi: 10.1038/s41467-021-23703-x (PMC8184813; doi:10.1038/s41467-021-23703-x)
Supplement: Supplementary file 1 — Supplementary Information [file 41467_2021_23703_MOESM1_ESM.pdf]

Supplementary Information for

A new high-voltage calcium intercalation host for ultra-stable and high-power calcium rechargeable batteries

Xu *et al.*

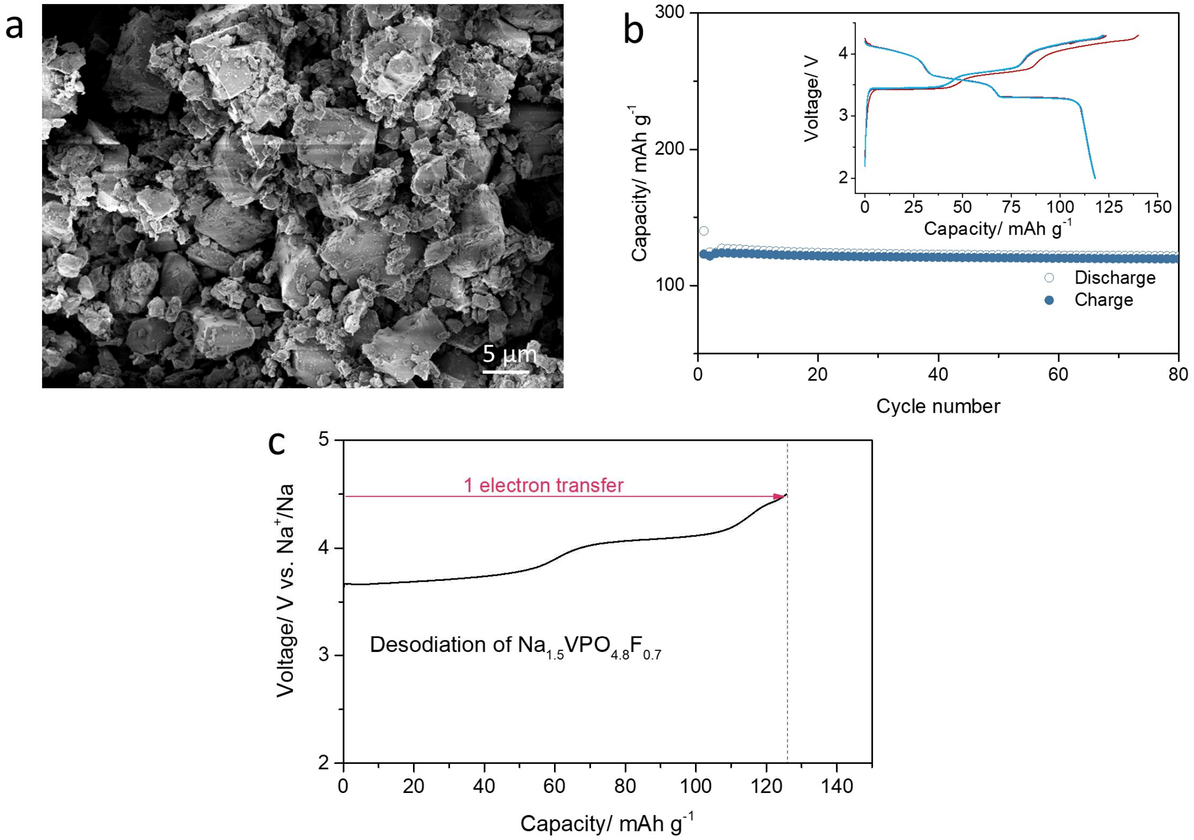

Supplementary Fig. 1 Morphological and electrochemical characterizations of $Na_{1.5}VPO_{4.8}F_{0.7}$. **a** SEM image of as-prepared $Na_{1.5}VPO_{4.8}F_{0.7}$ material with sizes of about 5 μm. **b** Cyclic capacities of $Na_{1.5}VPO_{4.8}F_{0.7}$ at 50 mA g^{-1} in Na-ion batteries with Na metal counter electrodes and 1M $NaPF_6$ EC/PC electrolyte, indicating its excellent stability in rechargeable batteries. **c** Extraction of Na ions from $Na_{1.5}VPO_{4.8}F_{0.7}$ to form $[]_{1.0}Na_{0.5}VPO_{4.8}F_{0.7}$ electrodes for CIBs.

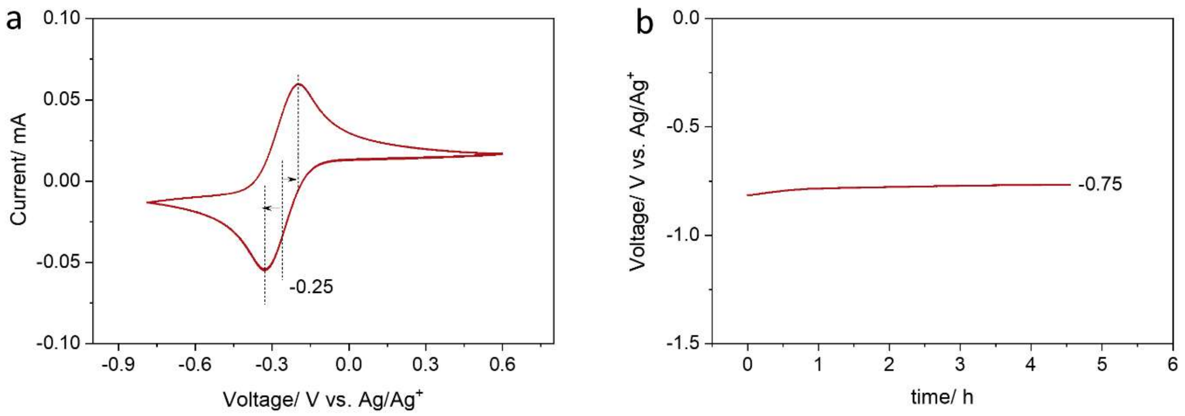

Supplementary Fig. 2 Ferrocene test to estimate activated-carbon voltage. **a** Cyclic voltammetry (CV) test in 0.5M $Ca(PF_6)_2$ EC/PC electrolyte containing 5 mM ferrocene with Pt as a working electrode and Ag/Ag^+ (0.01M $AgNO_3$) as a reference electrode. The scan rate is 1 mV s^{-1}. The Fc^+/Fc redox couple was measured at about -0.25 V vs. Ag/Ag^+, and the value of internal reference Fc^+/Fc against standard hydrogen electrode is 0.4 V. Thus, the Ag/Ag^+ reference voltage can be estimated to be 3.5 V vs. Ca/Ca^{2+}. **b** Open circuit voltage (OCV) of activated carbon electrode vs. Ag/Ag^+ reference electrode. The OCV is -0.75 V, thus the activated carbon electrode voltage can be estimated to be approximate 2.75 V vs. Ca/Ca^{2+}, consistent with previous report.[1]

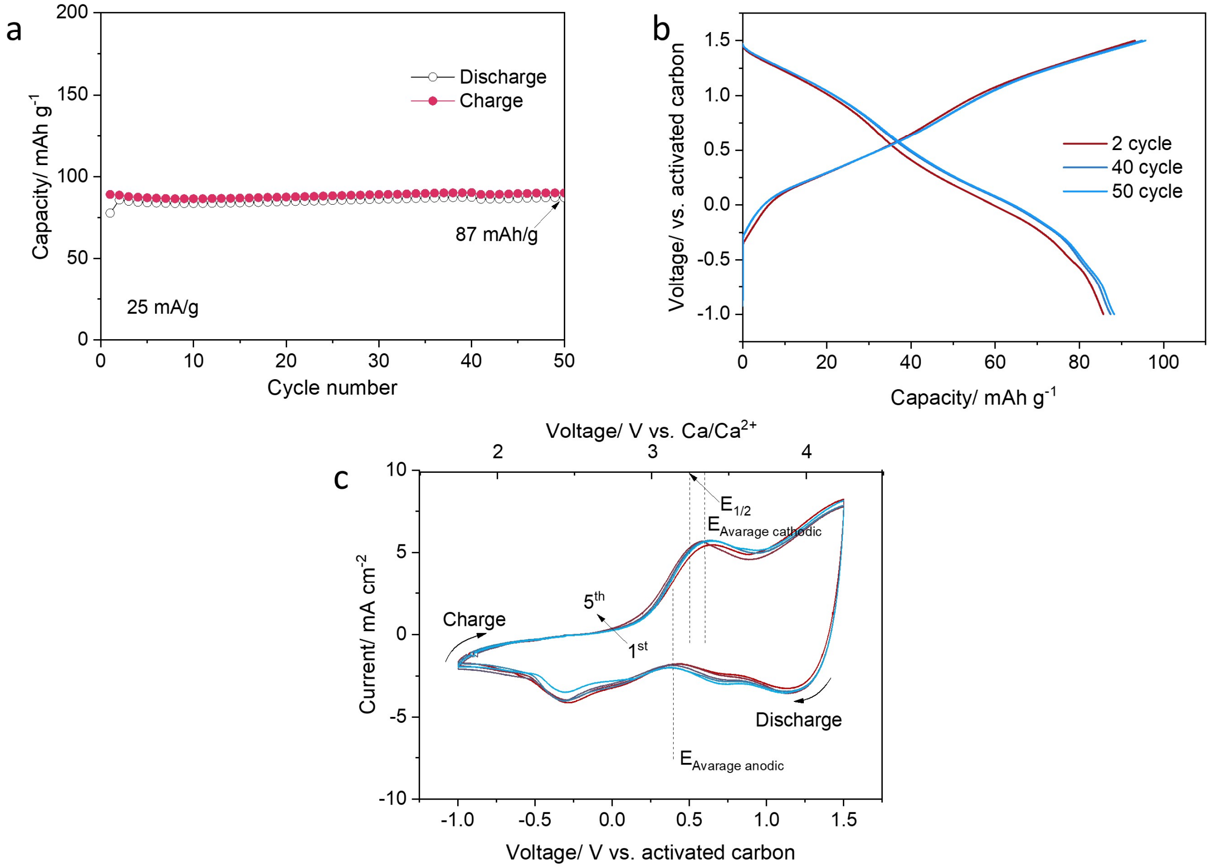

Supplementary Fig. 3 a Discharge/charge capacities of CIBs at 25 mA g^{-1}, a high capacity of 87 mAh g^{-1} was retained after 50 cycles. **b** The 2nd, 40th and 50th discharge/charge voltage profiles for the CIBs. **c** CV curves of CIBs at a scan rate of 0.2 mV s^{-1} between -1.0 and 1.5 V vs. activated carbon counter electrode. Overlap of CV curves indicates excellent reversibility of cathodes. The activated carbon is selected as both the counter and reference electrodes and the potential was estimated to be about 2.75 V vs. Ca/Ca^{2+}. We therefore calculated the average voltage for Ca insertion/extraction from NVPF to be approximate 3.2 V vs. Ca/Ca^{2+} in $Ca(PF_6)_2$ EC/PC electrolyte.

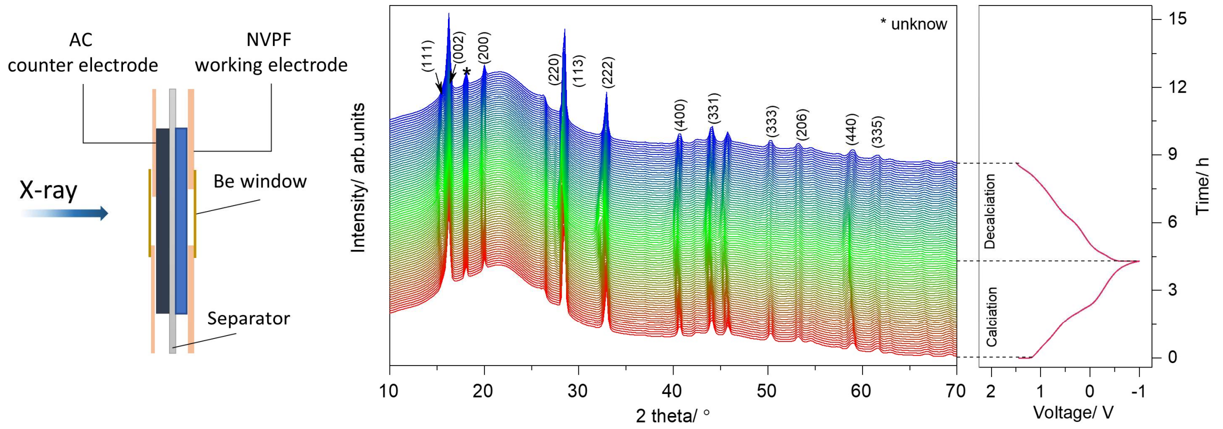

Supplementary Fig. 4 Schematic of the setup of synchrotron in-situ XRD experiment (left), the general in-situ XRD data (middle) and the corresponding discharge/charge voltage profile (right). Besides the interested peaks referring to NVPF cathode, the peak corresponding to the pouch cell component, Ca metal and some undefined signals are also presented.

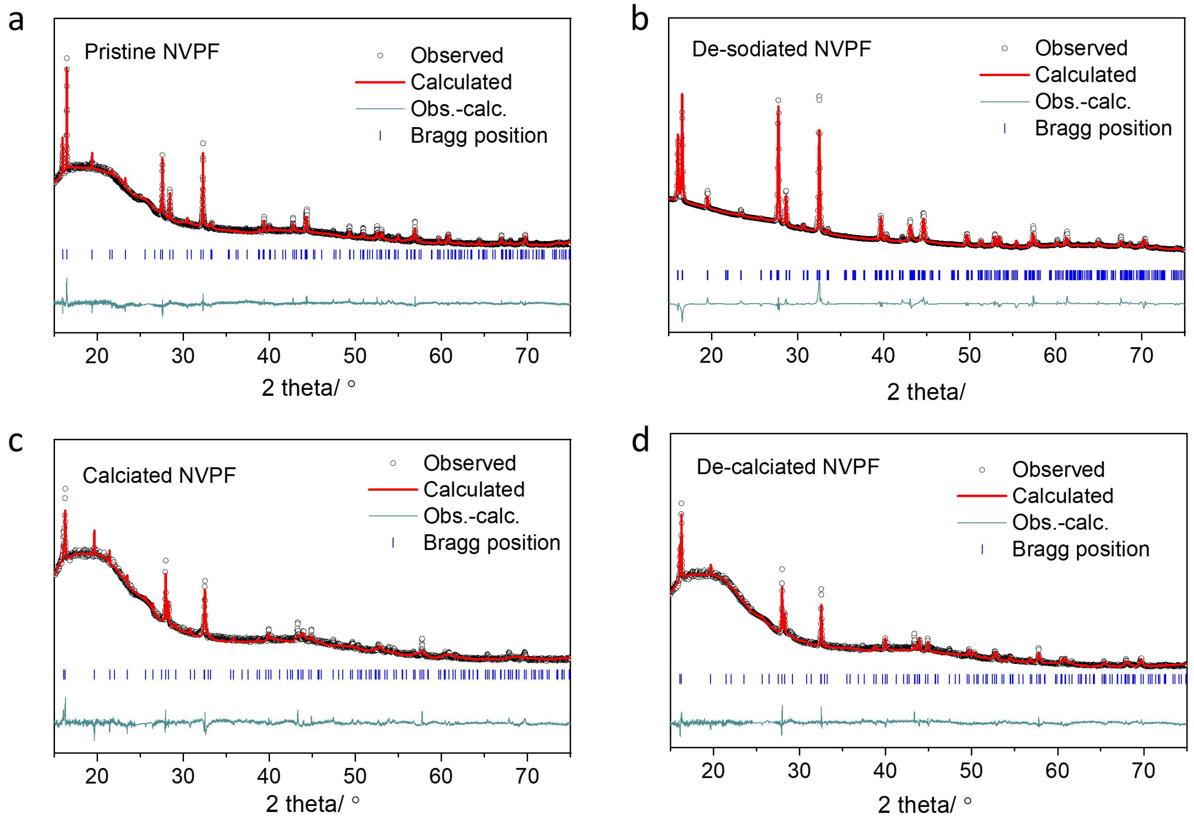

Supplementary Fig. 5 Rietveld refinement of the high resolution XRD patterns for **a** pristine NVPF, **b** desodiated NVPF, **c** calciated NVPF and **d** decalciated NVPF. The observed data points, calculated patterns, difference curve and Bragg positions are assigned to black dots, red lines, green lines and blue bars, respectively. The pristine NVPF could be well indexed with a space group of $P4_2/mnm$ in accordance with the previous report,[2] where VO_5F octahedra and PO_4 tetrahedra units construct a layer-like structure, and sodium ions are located at two different crystallographic sites, i.e. Na1 and Na2 sites as shown in the main manuscript (Fig. 3e).

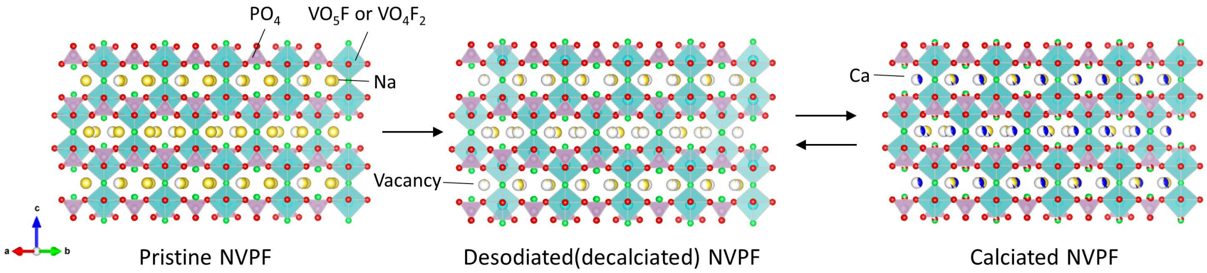

Supplementary Fig. 6 Schematic illustration of the structural evolution of NVPF for Ca ion storage. Pristine NVPF (left) was first charged to desodiated NVPF (middle). Then, Ca ions were inserted into the desodiated NVPF to form calciated NVPF (right). Reversible phase transition between calciated/decalciated NVPF happens during Ca insertion/extraction in following cycles in CIBs. Ca ions occupy the Na-vacancy layer between VO_5F, VO_4F_2/PO_4 layers. The overall framework is considered as a pseudolayered structure because cations form layers on the *ab* plane in the structure. The F, O, P, V, Na and Ca atoms are in color of green, red, pink, aqua, yellow and blue, respectively.

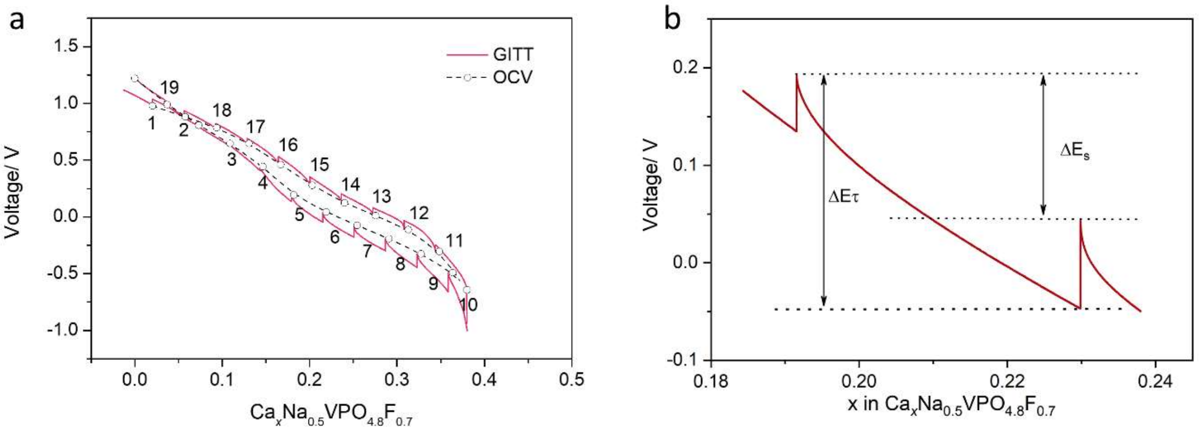

Supplementary Fig. 7 Galvanostatic intermittent titration technique (GITT) analysis of the NVPF cathode in CIB in discharging/charging mode at room temperature. **a** The GITT and open circuit voltage (OCV) curves, **b** the calculation method of Ca diffusion coefficient from GITT curve. The GITT was carried out after a single discharge/charge cycle by applying a constant current density of 10 mA g^{-1} for 30 min, followed by a relaxation potential measurement for 1 h. The OCV was used to calculate the Ca diffusion coefficient *D* as a function of *x* in $Ca_xNa_{0.5}VPO_{4.8}F_{0.7}$ according to the equation:[3,4]

$$D = \frac{4}{\tau\pi}\left(\frac{m_B V_m}{M_B S}\right)^2\left(\frac{\Delta E_s}{\Delta E_\tau}\right)^2$$

Where τ is duration of the galvanostatic discharge/charge, m_B, M_B and V_m refer to the active mass, the molar mass, and the molar volume of desodiated NVPF. S is the geometric surface area of the electrode, ΔE_s is the change of OCV values between two subsequent charge inject steps and ΔE_τ is the voltage variation during a current pulse. The change of Ca diffusion coefficient in desodiated NVPF with Ca content is plotted in Fig. 4a in the main context.

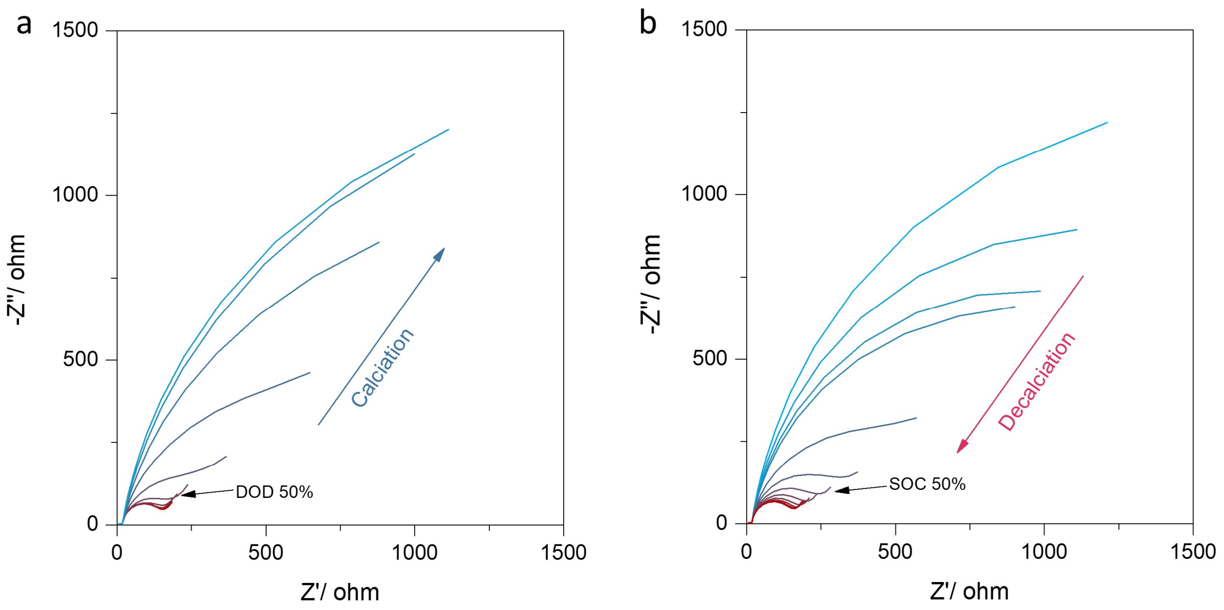

Supplementary Fig. 8 In-situ EIS measurement during a discharging/charging process of NVPF cathode. **a** EIS spectra during calciation (discharge), **b** EIS spectra during decalciation (charge). The Nyquist plots show that the R_{ct} (corresponding to the diameter of the semicircles at high frequency) are abruptly high in the late discharge stage (DOD of over 50%) and the early charge stage (SOC of below 50%), suggesting that the reaction kinetics were significantly limited as few Na vacancies were retained in the structure.

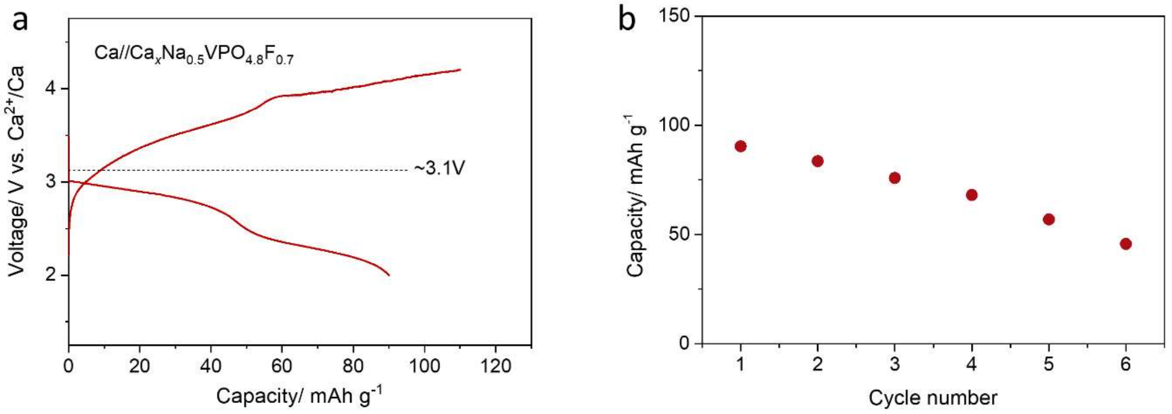

Supplementary Fig. 9 First five cycles of a primary Ca ion full cell consisting of Ca metal anode, desodiated NVPF cathode and $Ca(PF_6)_2$ EC/PC electrolyte. The reversibility of Ca metal stripping/plating process was ascertained under elevated temperatures (75-100 °C) in carbonate electrolyte, thus we cycled the cell at 75 °C.[5] The cell presented predominant discharge/charge plateaus, agree with those in desodiated NVPF//activated carbon cells (Fig. 1b), indicating successful Ca insertion/extraction. This reversible capacity and average voltage in this initial cycle is 85 mAh g^{-1} and 3.1 V, respectively, resulting an energy density of 263 Wh kg^{-1} based on cathode material. Aggressive capacity degradation was observed during cycles, possibly due to the instability of electrolyte or/and battery components under high temperature, requiring more efforts to develop stable Ca ion full cells in future studies.

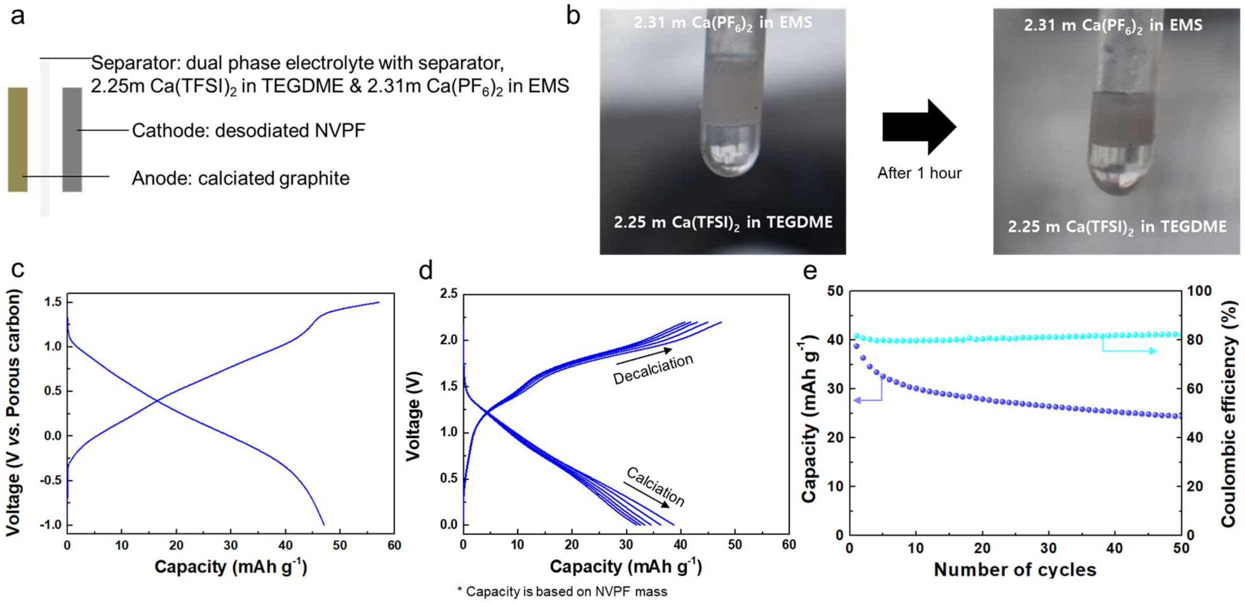

Supplementary Fig.10 An intercalation-based Ca-ion full cell performance. **a** Schematic of full cell configuration consisting of desodiated NVPF as cathode, calciated graphite as anode, and dual phase electrolyte. Graphite was first calciated in 1M $Ca(TFSI)_2$ in tetra ethylene glycol dimethyl ether (TEGDME) electrolyte.[6] **b** Immiscibility test for the dual phase electrolyte. After one-hour storage, two electrolytes remained unmixed, which implies our dual phase electrolyte system can be used to satisfy electrochemical compatibility of both active materials and electrolyte during cycling. The dual phase electrolyte is composed of $Ca(PF_6)_2$ in ethyl methyl sulfone (EMS) for the cathode and $Ca(TFSI)_2$ in TEGDME for the anode. **c** Galvanostatic profiles of desodiated NVPF cycled with porous carbon counter electrode and $Ca(TFSI)_2$ in EMS electrolyte. **d** Galvanostatic profiles of the full cell data for 6 cycles. **e** Cycle performance of the full cell, which shows reversible capacities over 50 cycles. The low coulombic efficiencies might be originated from the instability of interphase between electrolytes and/or active materials.

Supplementary Table 1 ICP-AES measured atomic ratios for pristine, desodiated, calciated, decalciated NVPF. The atomic ratios are normalized on base of vanadium content.

**Materials**	**Cation ratio**			
	**Ca**	**Na**	**V**	**P**
Pristine NVPF	0	1.48	1.00	1.00
Desodiated NVPF	0	0.55	1.00	1.00
Calciated NVPF	0.38	0.56	1.00	1.00
Decalciated NVPF	0.05	0.51	1.00	1.00

Supplementary Table 2 Comparison of the cyclic stability and rate capability of the current NVPF cathode and the peer electrodes for CIBs. CFR refers to capacity fading rate.

**Cathode materials**	**Operating voltage/ vs. Ca/Ca^{2+}**	**Cyclic stability**	**Rate capability**	**Ref.**
$Na_xMnFe(CN)_6$	~3.3	~60 mAh/g after 35 cycles at 10 mA/g	/	1
$VOPO_4{\cdot}2H_2O$	~2.8	86 mAh/g after 35 cycles at 20 mA/g, CFR: 0.57% per cycle	42.7 mAh/g at 0.2 A/g	7
α- MoO_3	~1.3	100 mAh/g after 12 cycles at 2 mA/g, CFR: 1.2% per cycle	/	8
$KFe^{3+}Fe^{2+}(CN)_6$	N	103 mAh/g after 80 cycles at 18 mA/g, CFR: 0.22% per cycle	~70 mAh/g at 0.072 A/g	9
$K_{0.25}MnO_2{\cdot}0.25H_2O$	~2.8	~50 mAh/g after 60 cycles at 25 mA/g, CFR: 1.12% per cycle	/	10
$NH_4V_4O_{10}$	N	130 mAh/g after 100 cycles at 100 mA/g	~50 mAh/g at 1A/g	11
$Ca_xNa_{0.5}VPO_{4.8}F_{0.7}$	~3.2	67 mAh/g after 500 cycles at 50 mA/g, CFR:0.02% per cycle	62 mAh/g at 0.1 A/g 43 mAh/g at 0.5 A/g	This work

Supplementary Table 3 Atomic positions for pristine NVPF from the high-resolution synchrotron XRD refinement. $\chi^2 = 4.6$.

**Label**	**Atom**	**Site**	**x**	**Y**	**z**	**Occupancy**
Na1	Na	8i	0.522(3)	0.216(9)	0	0.88(0)
Na2	Na	8i	0.82736	0.0512	0	0.62(0)
V1	V	8j	0.254(2)	0.254(2)	0.193(6)	1.0
P1	P	4d	0	0.5	0.25	1.0
P2	P	4e	0	0	0.259(0)	1.0
O1	O	16k	0.101(5)	0.412(7)	0.160(4)	1.0
O2	O	8j	0.111(3)	0.111(3)	0.198(0)	1.0
O3	O	8j	0.391(0)	0.391(0)	0.179(8)	1.0
O4	O	8j	0.248(1)	0.248(1)	0.363(7)	0.8
F1	F	4f	0.245(4)	0.245(4)	0	1.0
F2	F	8j	0.248(1)	0.248(1)	0.363(7)	0.2

Supplementary Table 4 Atomic positions for desodiated NVPF from the high-resolution synchrotron XRD refinement. $\chi^2 = 4.9$.

**Label**	**Atom**	**Site**	**x**	**y**	**z**	**Occupancy**
Na1	Na	8i	0.514(1)	0.234(5)	0	0.04(2)
Na2	Na	8i	0.803(0)	0.051(2)	0	0.46(1)
V1	V	8j	0.247(8)	0.247(8)	0.189(3)	1
P1	P	4d	0	0.5	0.25	1
P2	P	4e	0	0	0.255(3)	1
O1	O	16k	0.096(7)	0.407(7)	0.161(3)	1
O2	O	8j	0.094(7)	0.094(7)	0.136(0)	1
O3	O	8j	0.419(7)	0.419(7)	0.161(0)	1
O4	O	8j	0.248(6)	0.248(6)	0.347(3)	0.8
F1	F	4f	0.244(6)	0.244(6)	0	1.0
F2	F	8j	0.248(6)	0.248(6)	0.347(3)	0.2

Supplementary Table 5 Atomic positions for calciated NVPF from the high-resolution synchrotron XRD refinement. $\chi^2 = 6.2$.

**Label**	**Atom**	**Site**	**x**	**y**	**z**	**Occupancy**
Ca1	Ca	8i	0.523(4)	0.230(0)	0	0.30(0)
Na1	Na	8i	0.523(4)	0.230(0)	0	0.28(3)
Ca2	Ca	8i	0.803(0)	0.051(2)	0	0.08(2)
Na2	Na	8i	0.803(0)	0.051(2)	0	0.22(8)
V1	V	8j	0.248(0)	0.248(0)	0.189(3)	1.0
P1	P	4d	0	0.5	0.25	1.0
P2	P	4e	0	0	0.294(4)	1.0
O1	O	16k	0.119(2)	0.422(6)	0.140(5)	1.0
O2	O	8j	0.102(4)	0.102(4)	0.243(8)	1.0
O3	O	8j	0.390(3)	0.390(3)	0.224(5)	1.0
O4	O	8j	0.248(0)	0.248(0)	0.364(5)	0.8
F1	F	4f	0.240(6)	0.240(6)	0	1.0
F2	F	8j	0.248(0)	0.248(0)	0.364(5)	0.2

Supplementary Table 6 Atomic positions for decalciated NVPF from the high-resolution synchrotron XRD refinement. $\chi^2 = 5.4$.

**Label**	**Atom**	**Site**	**x**	**y**	**z**	**Occupancy**
Na1	Na	8i	0.509(1)	0.225(2)	0	0.28(1)
Na2	Na	8i	0.803(0)	0.051(2)	0	0.19(5)
Ca1	Ca	8i	0.509(1)	0.225(2)	0	0.01(2)
Ca2	Ca	8i	0.803(0)	0.051(2)	0	0.01(3)
V1	V	8j	0.24783	0.24783	0.18731	1
P1	P	4d	0	0.5	0.25	1
P2	P	4e	0	0	0.246(9)	1
O1	O	16k	0.105(9)	0.105(9)	0.238(8)	1
O2	O	8j	0.065(9)	0.376(9)	0.165(9)	1
O3	O	8j	0.397(3)	0.397(3)	0.166(7)	1
O4	O	8j	0.246(6)	0.246(6)	0.364(2)	0.8
F1	F	4f	0.240(6)	0.240(6)	0	1
F2	F	8j	0.246(6)	0.246(6)	0.364(2)	0.2

Supplementary references

1. Lipson, A. L. *et al.* Rechargeable Ca-ion batteries: a new energy storage system. *Chem. Mater.* **27**, 8442–8447 (2015).

2. Park, Y. U. *et al.* A new high-energy cathode for a Na-ion battery with ultrahigh stability. *J. Am. Chem. Soc.* **135**, 13870–13878 (2013).

3. Fattakhova, D., Kavan, L. & Krtil, P. Lithium insertion into titanium dioxide (anatase) electrodes: Microstructure and electrolyte effects. *J. Solid State Electrochem.* **5**, 196–204 (2001).

4. Wang, W. *et al.* Reversible structural evolution of sodium-rich rhombohedral Prussian blue for sodium-ion batteries. *Nat. Commun.* **11**, 980 (2020).

5. Ponrouch, A., Frontera, C., Bardé, F. & Palacín, M. R. Towards a calcium-based rechargeable battery. *Nat. Mater.* **15**, 169–172 (2016).

6. Richard Prabakar, S. J. *et al.* Graphite as a long-life Ca^{2+}-intercalation anode and its implementation for rocking-chair type calcium-ion batteries. *Adv. Sci.* **6**, 1902129 (2019).

7. Wang, J. *et al.* $VOPO_4 \cdot 2H_2O$ as a new cathode material for rechargeable Ca-ion batteries. *Chem. Commun.* **56**, 3805–3808 (2020).

8. Cabello, M. *et al.* Applicability of molybdite as an electrode material in calcium batteries: a structural study of layer-type Ca_xMoO_3. *Chem. Mater.* **30**, 5853–5861 (2018).

9. Kuperman, N. *et al.* High performance Prussian Blue cathode for nonaqueous Ca-ion intercalation battery. *J. Power Sources* **342**, 414–418 (2017).

10. Hyoung, J., Heo, J. W. & Hong, S. T. Investigation of electrochemical calcium-ion energy storage mechanism in potassium birnessite. *J. Power Sources* **390**, 127–133 (2018).

11. Vo, T. N., Kim, H., Hur, J., Choi, W. & Kim, I. T. Surfactant-assisted ammonium vanadium oxide as a superior cathode for calcium-ion batteries. *J. Mater. Chem. A* **6**, 22645–22654 (2018).
